# Self-assembled three-dimensional and compressible interdigitated thin-film supercapacitors and batteries

**DOI:** 10.1038/ncomms8259

**Published:** 2015-05-29

**Authors:** Gustav Nyström, Andrew Marais, Erdem Karabulut, Lars Wågberg, Yi Cui, Mahiar M. Hamedi

**Affiliations:** 1 Department of Fibre and Polymer Technology, and Wallenberg Wood Science Centre, KTH Royal Institute of Technology, School of Chemical Science and Engineering Teknikringen 56, 10044 Stockholm, Sweden; 2 Department of Materials Science and Engineering, Stanford University, 476 Lomita Mall, McCullough 343, Stanford, California 94305, USA

## Abstract

Traditional thin-film energy-storage devices consist of stacked layers of active films on two-dimensional substrates and do not exploit the third dimension. Fully three-dimensional thin-film devices would allow energy storage in bulk materials with arbitrary form factors and with mechanical properties unique to bulk materials such as compressibility. Here we show three-dimensional energy-storage devices based on layer-by-layer self-assembly of interdigitated thin films on the surface of an open-cell aerogel substrate. We demonstrate a reversibly compressible three-dimensional supercapacitor with carbon nanotube electrodes and a three-dimensional hybrid battery with a copper hexacyanoferrate ion intercalating cathode and a carbon nanotube anode. The three-dimensional supercapacitor shows stable operation over 400 cycles with a capacitance of 25 F g^−1^ and is fully functional even at compressions up to 75%. Our results demonstrate that layer-by-layer self-assembly inside aerogels is a rapid, precise and scalable route for building high-surface-area 3D thin-film devices.

## Abstract (web summary)

It is challenging to construct three-dimensional thin-film energy-storage devices. Here the authors present supercapacitors and batteries based on layer-by-layer self-assembly of interdigitated thin films inside aerogels, demonstrating energy storage as well as compressibility in three-dimensional devices.

## Graphical abstract

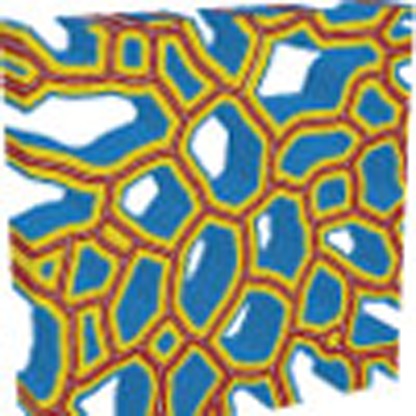

The concept of storing charge in three-dimensional (3D) architectures has been pursued for more than a decade^1^. Such devices can however only be built by patterning complex interdigitated structures of different thin film materials inside a bulk volume with a high surface area and this has been a highly challenging and unresolved problem to date^1^. Even though recent advances have been made with interdigitated two-dimensional (2D) devices or semi-3D devices^2^,^3^,^4^,^5^, only a few examples of full 3D energy-storage devices have been shown^6^,^7^,^8^,^9^,^10^,^11^,^12^. Most of the proposed designs are limited by the etching, micro fabrication or 3D printing techniques used to build up the 3D structure^6^,^7^,^8^,^10^,^13^. Simpler and more scalable designs have been suggested by building the device around a carbon or copper foam structure^11^,^12^. Although functioning, these latter devices show low rate performance as a result of backfilling the structure with the second electrode and thereby creating long ionic and electronic transport pathways.

So far, none of these technologies have emerged as ideal and the advantages of 3D batteries predicted by simulations^14^ remain to be experimentally verified. The three main shortcomings of the current 3D technologies are as follows: (1) the complexity of the design usually limits the size of the 3D devices and the process is non-scalable with size; (2) low surface-area substrates are usually employed, which in turn limit the gained area factor of the devices compared with traditional 2D devices; and (3) the construction methods are limited to a small class of materials.

Herein we present a facile method to build fully interdigitated 3D energy-storage devices, by using layer-by-layer (LbL) assembly to self-assemble the entire device inside of an aerogel. The method presented is applicable to a range of different materials and offers highly precise conformal depositions with nanometre thickness control of the assembled layers. Aerogels have the highest specific surface area among any man-made material and can be formed from a variety of different inorganic and organic materials^15^,^16^, their pore volume and mechanical properties can be finely tuned and they can be prepared with different form factors. This work has the potential to expand the range of electronic devices by making it possible to produce macroscopic compressible, bulk thin-film devices, with a variety of functions (for example, soft, insulating, or shock-absorbent electronics, gas and chemical bulk sensors, volumetric light emission and fuel cells).

## Results

### 3D supercapacitor assembly

In this study, LbL self-assembly was used to assemble complete 3D supercapacitors and hybrid batteries onto anionically charged (2.3 mmol g^−1^ carboxyl groups) cross-linked cellulose nanofibril (CNF) aerogels^17^. These materials have an open-cell foam structure allowing liquids to pass through the structure (a prerequisite for the LbL assembly) and combine high mechanical strength (0.1 MPa compressive Young's modulus) with flexibility, a large surface area (up to 600 m^2^ g^−1^) and chemical inertness, enabling them to work in most solvents (including ethanol, acetone and toluene)^17^,^18^,^19^. The LbL method, compatible with aqueous and non-aqueous media^20^, is based on the adsorption of oppositely charged species dispersed in solutions that flow through the framework^17^. This process is different from traditional diffusion-limited LbL systems, as the convective nature of the flow allows each layer to be deposited in less than 1 s. Furthermore, the assembly method does not scale in speed with an increasing device size. This is important for upscaling of the device. In practice, the complete self-assembly process takes 1 s for each layer, and thus with a maximum of hundred layers the result is a total assembly time of only a few minutes, irrespective of the device size.

**Figure 1:**
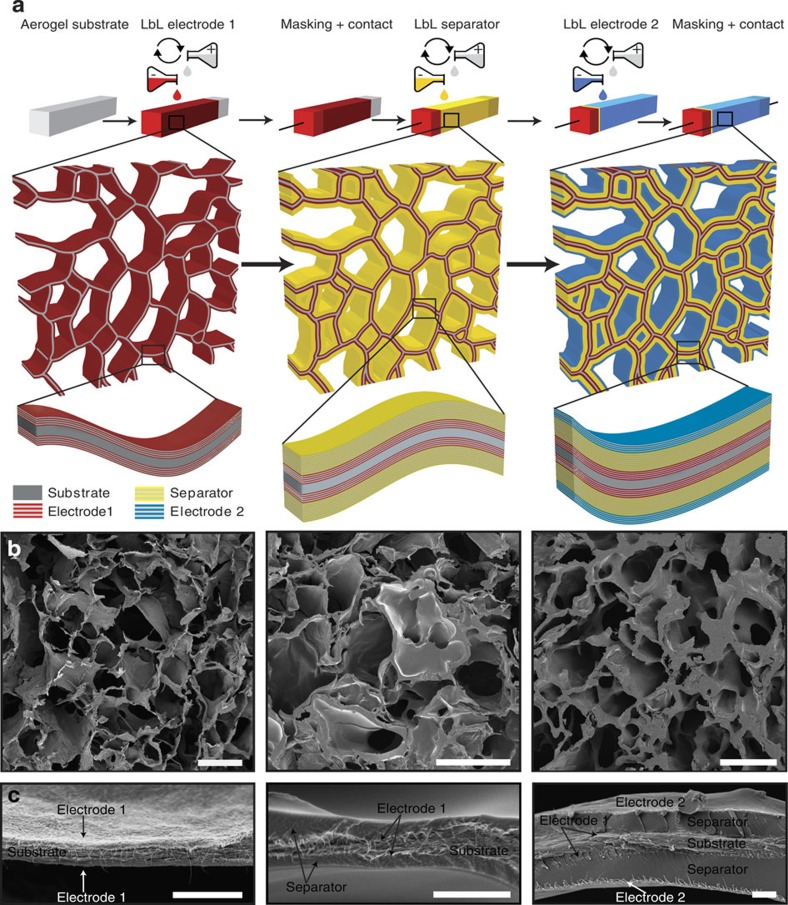
Device assembly and structural architecture. (**a**) Schematics of the LbL process used to assemble 3D devices in an aerogel and (**b**,**c**) cross-section SEM images of the first PEI/CNT electrode (left column), the PEI/CNT electrode with separator (middle column) and the full device (right column). Scale bars, (**b**) 50 μm and (**c**) 2 μm.

To show the concept of a fully integrated 3D electrochemical device, we chose a carbon nanotube (CNT)-based supercapacitor operating in an aqueous electrolyte. The devices were built using LbL self-assembly, where each layer was sequentially assembled on the walls of the aerogel in the bulk of the material as shown in the schematic Fig. 1a and in the scanning electron microscopy (SEM) images in Fig. 1b,c. The electrodes were constructed from interchangeable layers of cationic polyethyleneimine (PEI) and anionic (–COOH functionalized) single-wall CNTs (five bilayers, Supplementary Fig. 1). A PEI/polyacrylic acid (PAA) multilayer film (30 bilayers) served as a separator. Paraffin wax was used as a mask to protect the electrical contacts against side reactions with the electrolyte and to effectively define the active device volume (Supplementary Fig. 2).

The supercapacitor (three layers) is seen on both sides of each pore wall, that is, the full device is a seven-layer structure, including the aerogel substrate wall. The lower-resolution SEM images show the gradual thickening of the pore walls when the first electrode, the separator and the second electrode were added onto the pristine aerogel. In the higher magnification images, the thickness of the CNT electrodes (∼100–200 nm) and of the separator (∼1–2 μm) can be estimated (Supplementary Fig. 3). The amount of CNT deposited in each of the electrodes was previously determined to be 2.8±0.2 wt% (ref. 17), giving a total CNT content of 5.4 wt% for the full device. The bulk electronic conductivity of the CNT electrode was measured to be 9.8 × 10^−2^ S cm^−1^.

### 3D supercapacitor electrochemical characterization

**Figure 2:**
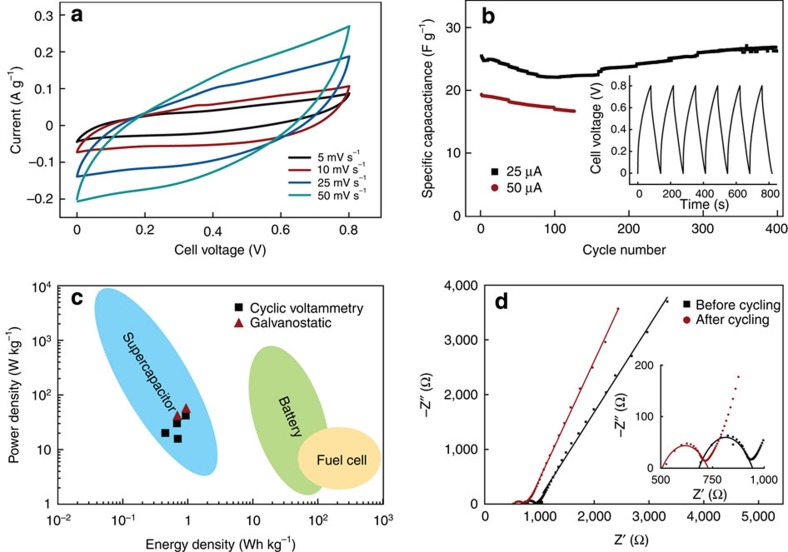
3D supercapacitor characterization. (**a**) CVs at different scan rates and galvanostatic cycling (**b**, inset) of the device are shown together with (**b**) life-cycle performance, (**c**) Ragone plot and (**d**) electrochemical impedance spectroscopy data. The Ragone plot is based on the cyclic voltammetry (squares) and the galvanostatic data (triangles). Aqueous 1 M Na_2_SO_4_ pH 7 was used as electrolyte in all experiments.

Figure 2a shows cyclic voltammograms (CVs) of the interdigitated 3D device in aqueous 1 M Na_2_SO_4_ pH 7 electrolyte at different scan rates and reveals the typical square-shaped voltammograms resulting from double-layer charging of a carbon surface. It should be noted, however, that a high device resistance, sometimes observed as a result of poor electrode contact or the use of too thick separators, might lead to tilted CV curves. The constant current charge and discharge curves (Fig. 2b, inset) also reflect the capacitive behaviour seen in the voltammograms with a linear dependence of the cell voltage as a function of time. As our results represent the first demonstration of a true 3D supercapacitor^21^, a direct comparison with previous 3D devices in the literature is difficult^7^,^9^,^11^. The characteristic features of the electrochemical charging and discharging curves are however similar to those obtained for traditionally assembled 2D devices using the same active materials^17^. This indicates good electronic transport within the interdigitated network as well as good ionic mobility across the multilayer separator.

An electrode-specific capacitance of 25 F g^−1^ was calculated from the slope of the constant current discharge curve, based on the mass of active material and assuming a current corresponding to a 60 C (1 min) discharge rate (Fig. 2b). Although this capacitance value is lower than some recently reported values^17^,^22^, it is still comparable to values from other traditional carbon-based supercapacitors^23^, placing it within the region of standard supercapacitors on the Ragone plot (Fig. 2c). The capacitance value obtained is also an indication that a large part of the available surface area within the bulk 3D volume of the device is available for charge storage. Considering the complexity of this self-assembled 3D device with its fully integrated structure this is a remarkable result and a great improvement on to the 3D devices so far reported in the literature^6^,^7^,^8^,^9^,^10^,^11^,^12^.

The devices showed a stable cycling behaviour at the 60 C rate for 400 cycles after which the charging rate was increased to 160 C (22 s). At this higher rate, 75% of the initial capacitance was maintained and the devices again showed a stable performance (Fig. 2c). These data show that the separator effectively prevented short-circuiting between the anode and cathode, and emphasizes the power of the LbL self-assembly technique for creating pinhole-free separators with complex 3D geometries. After cycling the device, the Nyquist curve (Fig. 2d) shifted towards lower resistance values and a narrowing of the high-frequency semicircle was observed. This indicates a decrease in the ionic electrolyte resistance. Altogether, the results shown here represent the first realization of a fully integrated true 3D supercapacitor device^21^,^24^.

### 3D supercapacitor compressibility

**Figure 3:**
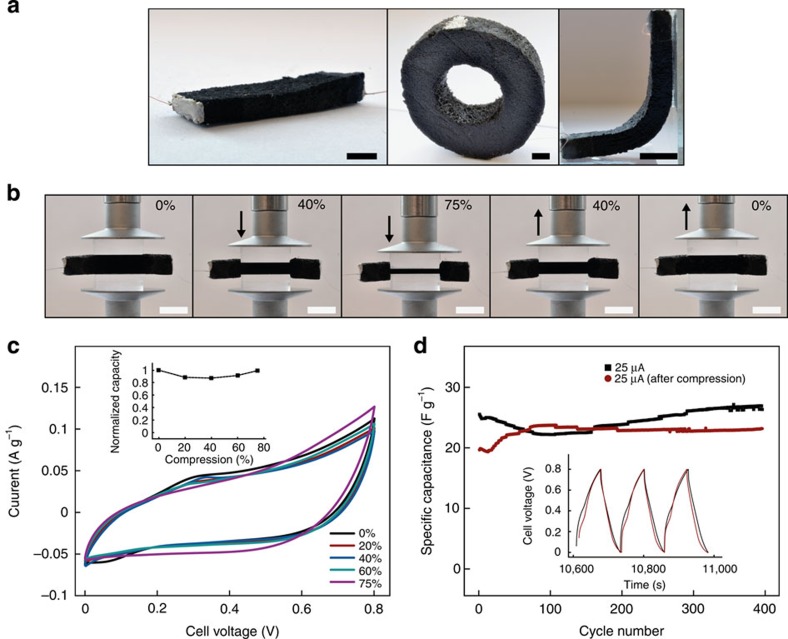
Supercapacitor shape flexibility and operation under compression. (**a**) Photographs of rectangular and donut-shaped devices, as well as of the device during bending, and (**b**) 0%–75% reversible compression cycles. Scale bars, 0.5 cm. (**c**) CVs performed *in situ* during compression at a scan rate of 10 mV s^−1^ with corresponding calculated normalized charge capacities (c, inset). (**d**) Comparison of the galvanostatic curves before and after compression of the device (inset) and cycle life data before and after compression. Aqueous 1 M Na_2_SO_4_ pH 7 was used as electrolyte.

One of the advantages of the sponge-like architecture of the CNF aerogel is its inherent flexibility in compression and bending. This flexibility may prove to be important to accommodate stresses related to the volume expansion of electroactive materials during operation^25^. It also opens up new possibilities for flexibility on the device level. Different non-cubic geometries can be realized (Fig. 3a). In addition, the devices are bendable and compressible, and, to exploit this flexibility, experiments were performed where the device was operated under bending and compression (Fig. 3a,b). The device could be reversibly bent to 90° or compressed up to 75% without any observable structural damage. Further and even more remarkable, during bending and compression, the *in-situ* CV showed only minor changes in the device performance (Fig. 3c and Supplementary Fig. 4) and in the available charge capacity (Fig. 3c, inset). Prolonged galvanostatic cycling also showed that the performance of the device over time was unaffected by the aerogel compression (Fig. 3d).

### Copper hexacyanoferrate cathode assembly and characterization

**Figure 4:**
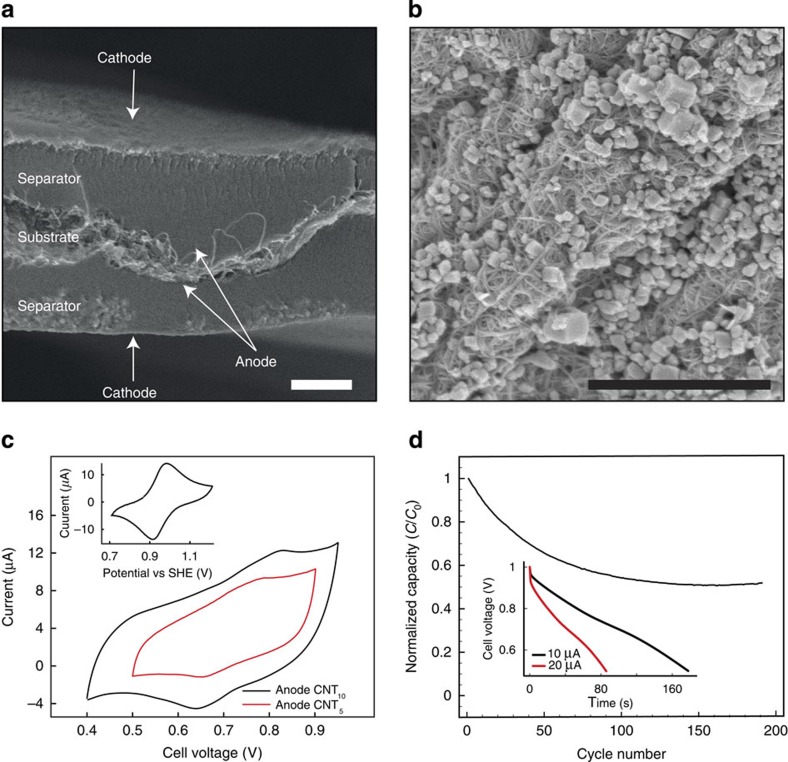
Copper hexacyanoferrate cathode and 3D hybrid battery characterization. (**a**) Cross-section SEM images of the copper hexacyanoferrate/CNT 3D hybrid battery and (**b**) SEM image of the copper hexacyanoferrate cathode top surface. Scale bars, 1 μm. (**c**) CVs for the 3D hybrid battery and for the cathode (inset) five bilayers (red line) and ten bilayers (black line) of PEI/CNT in the anode and three bilayers of PEI/CNT and four bilayers of PEI/CuHCF in the cathode (both devices). (**d**) Galvanostatic charging and discharging of the CNT_10_ device (inset) and zoom in on one discharge cycle using 10 (black line) and 20 μA (red line) charging current and device cutoff voltage 0.5 and 1 V, respectively. The scan rate was 1 mV s^−1^ in all CV experiments and aqueous 1 M KNO_3_ 0.01 M HNO_3_ was used as electrolyte in all experiments.

To extend this concept towards a full electrochemical cell based on redox reactions, an ion-insertion electrode was assembled. Copper hexacyanoferrate (Cu^II^−N≡C−Fe^III/II^), an open-framework material with the Prussian Blue crystal structure, was chosen as a cathode^26^,^27^,^28^ and aqueous 1 M KNO_3_ 0.01 M HNO_3_ was used as an electrolyte. Copper hexacyanoferrate is a negatively charged and water-dispersible nanoparticle (Supplementary Fig. 5) and is therefore compatible with the LbL assembly. These nanoparticles were LbL assembled interchangeably with PEI around a CNT conductive layer to give the final cathode structure as (PEI/Cu^II^−N≡C−Fe^III/II^)_3_(PEI/CNT)_3_(PEI/Cu^II^−N≡C−Fe^III/II^)_3_. As seen in the SEM images (Fig. 4b), Cu^II^−N≡C−Fe^III/II^ formed homogeneous layers of cubic nanoparticles on the surface of the CNT-coated aerogel. In the presence of K^+^, the Cu^II^−N≡C−Fe^III/II^ forms a single-phase insertion reaction:^26^


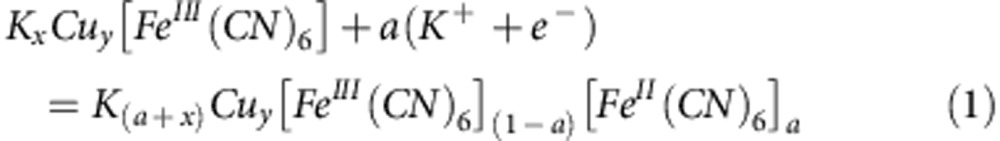


This reaction has a standard potential of ∼1 V versus the standard hydrogen electrode , making it suitable for use as a cathode in aqueous electrolytes. In the CV of the cathode (Fig. 4c, inset), highly reversible oxidation and reduction peaks were seen, in agreement with previous findings^26^,^27^, overlaid on the capacitive background current from the double-layer charging of the CNTs. By integrating the charge from the CV, a specific capacity of 7.2 mAh g^−1^ was obtained. This is lower than the theoretical specific value and may be partially explained by the presence of zeolitic water in the Cu^II^−N≡C−Fe^III/II^ structure^26^ as well as by uncertainties in the quantification of the amount of Cu^II^−N≡C−Fe^III/II^ in the electrode. The electrode showed a stable performance over several days of testing with no evident loss of capacity, indicating that the LbL-assembled electrode is stable, and that Cu^II^−N≡C−Fe^III/II^ nanoparticles do not detach or dissolve in the electrolyte.

### 3D hybrid battery assembly and characterization

A 3D hybrid battery was LbL assembled following the methodology described in Fig. 1, combining the Cu^II^−N≡C−Fe^III/II^ cathode with (PEI/PAA)_30_ as the separator and with (PEI/CNT)_5,10_ as the anode. In the cross-section of one of the pores of the device (Fig. 4a), the structure of the Cu^II^−N≡C−Fe^III/II^ particles is clearly seen (outer surface) as well as the CNTs pulled out from the anode on either side of the aerogel pore wall separated by the (PEI/PAA)_30_ LbL system (∼1–2 μm). The peaks corresponding to the redox reaction of the Cu^II^−N≡C−Fe^III/II^ were also found in the CV for the full device (Fig. 4c). From the CV, specific charge capacities of 5.3 and 5.9 mAh g^−1^ were obtained for devices with five and ten CNT bilayer anodes, respectively. These values are close to the specific capacity for the cathode and indicate an almost complete utilization of the Cu^II^−N≡C−Fe^III/II^ in the device

The device was further characterized by galvanostatic charging and discharging, and was operating for 190 cycles (with a 52 % capacity retention) after which the testing was terminated (see Fig. 4d). When increasing the charging current from 10 to 20 μA corresponding to a change in charging rate from 20 to 42 C, only a small decrease (∼5 %) in the available capacity was found (see Fig. 4d, inset). This is similar to previous results with copper hexacyanoferrate^24^ and indicates that the particles are capable of fast charging and discharging, and that the performance of the active material is not limited by the present device design.

## Discussion

The LbL-assembled electrodes were highly stable in the electrolyte. We saw no signs (drastic loss of capacity) indicating delamination of active materials during operation. The fact that the devices were fully dried following assembly could also mean that amide bonds were formed between primary amines of the PEI and carboxylic acid groups of the functionalized CNTs, improving both the adhesion to the substrate and the adhesion within the electrodes^29^. Furthermore, as LbL films can be formed and are stable at ionic strengths exceeding those used here in the electrolyte^30^,^31^, the electrodes should remain stable during device operation. Considering the good stability of CNT in supercapacitors^32^ and the versatility of the LbL-based method, enabling the use of many different materials, further optimization of the device life cycle should also be possible.

It has previously been demonstrated that the ionic conductivity across polyelectrolyte films (similar in nature to the separator used here) can change by several orders of magnitude as a result of hydration^33^,^34^, and this conductivity change was not instantaneous but occurred over a time period of at least 1 h. During the preparation of the present devices, the polyelectrolyte separator was fully dried. Before electrochemical testing, the devices were re-wetted with the electrolyte and the first Nyquist plot was recorded. The second Nyquist plot was collected following about 24 h of device operation. After this time we assume that the polyelectrolyte separator is fully equilibrated with the electrolyte, and that the ionic conductivity across the separator has increased and stabilized to the maximum conductivity that can be achieved based on the ionic conductivity of the electrolyte. This change in ionic conductivity across the separator may explain the observed shift in the Nyquist plot (see Fig. 2d).

To rationalize the result of the device operation during compression, we believe that it is mainly the voids in the device that are being compressed. If the entire pores were locally fully compressed so that two pore walls come into contact, this would not result in a short circuit, as the outer layers of the two surfaces are from the same electrode (see Fig. 1). Furthermore, the structural design of the open-cell network provides an effective strategy for allowing extreme compression, while microscopically translating the large deformations to small local strains, preventing fracture of the active materials. These combined properties thus define a unique class of devices that are soft and highly resilient to compression.

The drop in capacity seen during cycling of the 3D hybrid battery may be explained by a partial degradation of the copper hexacyanoferrate particles. Prolonged galvanostatic cycling of the copper hexacyanoferrate electrode in a three-electrode setup indicates that the expected drop in capacity based on degradation over 200 cycles should be much <48% (see Supplementary Fig. 6). The three-electrode data further indicate that the copper hexacyanoferrate cathode is relatively stable as shown by >800 cycles with a capacity retention of 67%. Furthermore, there is no indication in the data that the capacity fade should be caused by delamination or detachment of active particles from the LbL assembly. Another possible explanation of the loss of device capacity may be a drift in electrode potential of the copper hexacyanoferrate cathode as a result of the electrodes not being perfectly mass balanced. This could cause an apparent loss of capacity in the cell.

In conclusion, we have shown that fully interdigitated 3D supercapacitors and batteries can be self-assembled inside high-surface area aerogels using a rapid and scalable methodology. The obtained devices show stable operation without short-circuiting, are bendable, compressible and can be made with arbitrary form factors. These results are very promising and show that this LbL-based methodology can produce fully interdigitated 3D devices with a complex structure containing a variety of materials. The concept presented here therefore has the potential to be extended to other materials and other types of 3D devices^35^.

## Methods

### Materials

Branched PEI (60 kDa) was purchased from Acros Organics and 1,2,3,4 Butanetetracarboxylic acid (BTCA), sodium hypophosphite (SHP) and PAA (240 kDa) were purchased from Sigma Aldrich. All chemicals were used without further purification. Single-wall CNTs functionalized with carboxyl groups (P3-SWCNT) were from Carbon Solutions. Copper hexacyanoferrate was synthesized according to a previously described co-precipitation method^26^,^27^. nanocellulose were provided as a gel, with 1.84 wt% of nanocellulose in water, by Innventia AB, based on production methods described in ref. 36.

### Material preparation for LbL

PEI and PAA were used at a concentration of 1 g l^−1^ with a pH of 10 and 5, respectively. The carboxyl functionalized SWCNTs were dispersed in deionized water with an initial concentration of 1 g l^−1^, using an ultrasonic probe (VCX 750, Sonics & Materials Inc.) for 30 min. The exfoliated CNTs were further centrifuged at 20,000*g* for 1 h (to remove larger aggregates) followed by decanting of the supernatant, which was used for the further experiments. The copper hexacyanoferrate was dispersed in water at pH 7 and sonicated to liberate the particles. A concentration of 19 g l^−1^ was used for the LbL.

### Fabrication of wet-resilient aerogels

BTCA and SHP were mixed in the nanocellulose gel as powder at a 1:1 mass ratio (BTCA) and 2:1 mass ratio (SHP), followed by 15 min of stirring using an Ultra Turrax T25 (IKA, Germany), at 10,000 r.p.m. The gel was subsequently frozen in aluminum forms using liquid nitrogen and then freeze dried. Finally, the freeze-dried aerogel was heated to 170 °C for 5 min, to permanently cure the ester cross-links. All aerogels were thoroughly rinsed with Milli-Q water after the cross-linking, to ensure that the residuals from the cross-linking were washed out.

### Supercapacitor build-up

First, a cross-linked wet-stable nanocellulose aerogel was cut into the desired shape using a razor blade. The LbL deposition method was then used to assemble the first electrode using a previously described rapid filtration procedure^17^. PEI was introduced as an anchoring layer for the subsequent build-up of a total of five bilayers of anionic (−COOH functionalized) CNTs/PEI (see Supplementary Fig. 1). To minimize the risk of short-circuiting, one edge of the specimen was left without CNT coating. The CNT-coated aerogel was thereafter dried in the oven at 100 °C for 1 h before contacting the first electrode using a conductive silver paint and a thin copper wire. The contact point was protected by paraffin wax, which prevented electrolyte from reaching the contacts during device operation.

To separate the electrodes, a 30-bilayer film of (PEI/PAA) was assembled using the same LbL filtration setup. PEI and PAA dispersions (1 g l^−1^) were used without adjusting the pH and the sample was inverted after 10, 20 and 25 bilayers, to ensure homogenous film formation.

The second electrode was assembled in a similar manner as the first electrode, using five bilayers of PEI and COOH-functionalized SWCNTs. The device was thereafter left to dry under ambient conditions overnight before contacting the second electrode and sealing the contact with paraffin wax. As the two waxed sides completely block the electrolyte (see Supplementary Fig. 2), the interdigitated volume of the device can be defined as the non-waxed volume of the aerogel. The typical dimensions of the active volume of the device were ∼13 × 3 × 7 mm^3^.

### Copper hexacyanoferrate cathode build-up

The copper hexacyanoferrate cathode was built by first assembling the three bilayers of PEI and copper hexacyanoferrate. This was followed by three bilayers of PEI/CNT to increase the conductivity of the electrode. Finally, another three-bilayer PEI and copper hexacyanoferrate was built. All LbL steps were performed without intermediate drying. Before testing, the electrode was allowed to dry under ambient conditions.

### Battery build-up

A similar LbL procedure as that used to assemble 3D supercapacitors was used to also assemble 3D hybrid batteries. (PEI/CNT)_5_ and (PEI/CNT)_10_ were used as anodes, (PEI/PAA)_30_ was used as separator and (PEI/CuHCF)_2_(PEI/CNT)_3_(PEI/CuHCF)_2_ was used as cathode. The anode and separator were formed and contacted the same way as for the supercapacitor. The cathode was formed similarly as described above. After assembling the cathode, the device was dried under ambient conditions and the cathode was contacted and waxed similarly as the anode.

### Determination of CNT and copper hexacyanoferrate mass

For CNTs, Beer–Lambert's law *A*=*ebc* (where *A* is the optical density, *e* the extinction coefficient, *b* the path length and *c* the concentration) was used to determine the weight of the LbL-adsorbed CNT. First, the extinction coefficient *e* was determined by dispersing CNTs of varying concentrations in water. The actual concentrations of the different CNT dispersions were determined by measuring the dry content weight of the dispersion. The measured concentrations based on dry weight were plotted against the ultraviolet–visible absorption at 500 nm and a linear calibration curve in agreement with the Beer–Lambert's law was used to calculate *e* to 17.9 l g^−1^ cm^−1^. The absolute weight of the adsorbed CNT on the aerogel was then calculated by measuring the weight of the CNT in the residue liquid that had passed the aerogel (using ultraviolet–visible and Beer's law) and subtracting this weight from the initial weight.

An alternative second method was also used by measuring the dry weight of the aerogel before and after deposition of the first (PEI/CNT) electrode layers. The dry weight was obtained by 1 day of drying at 170 °C, to omit the additional weight of water. The weight was approximately measured to 3 wt%.

For the copper hexacyanoferrate cathode and hybrid battery, the amount of Cu was quantified with elemental analysis and renormalized to the full complex weight using the empirical chemical formula: CuC_6_FeN_6_. This weight for the full copper hexacyanoferrate complex was used to normalize the specific charge capacities presented in the main text.

### Electrochemical characterization

Aerogel morphology and LbL coatings were investigated by SEM (Hitachi S-4800) operated at 1 kV. A 5-nm gold palladium coating was applied to minimize charging effects. Electrical resistance measurements were performed using a Keithley 2,400 source meter. An Autolab PGSTAT302N was used for the electrochemical characterization. Cyclic voltammetry was performed using scan rates of 5–50 mV s^−1^ in a two-electrode setup. Constant current charging and discharging was done using currents between 25 and 200 μA, corresponding to current densities between 0.07 and 0.5 A g^−1^ based on the total carbon mass of the device. The voltage window was 0–0.8 V. The specific cell capacitance (in F g^−1^) was calculated from the slope of the discharge curve using the following equation: 
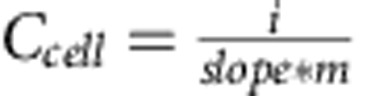
, where *i* is the discharge current (in A), slope is the linear fit to the discharge curve (in V s^-1^) and *m* is the total active mass of CNT in the device (in g). To get the single electrode-specific capacitance the following expression for two capacitors in series is used: 
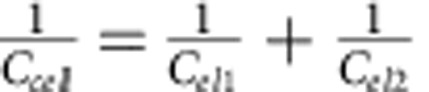
, and using that the capacitance of electrode 1 and electrode 2 is equal: *C*_el_=2*C*_cell_^29^. Renormalizing with the mass of a single electrode introduces another factor 2, to give the final expression that was used to calculate the single electrode-specific capacitance as: *C*_el,specific_=4*C*_cell_. Electrical impedance spectroscopy was performed in the frequency range 1 MHz to 0.1 Hz around the open-circuit potential of the device (close to 0 V) using an AC voltage amplitude of 5 mV. Aqueous 1 M Na_2_SO_4_ pH 7 was used as electrolyte for the supercapacitor measurements and aqueous 1 M KNO_3_ 0.01 HNO_3_ was used for the copper hexacyanoferrate cathode and the hybrid battery measurements. For measurements under compression, a custom-made setup using a digital caliper was used and the scan rate in the *in-situ* CVs was 10 mV s^−1^. The copper hexacyanoferrate cathode (standard three-electrode setup) and hybrid 3D batteries were tested using cyclic voltammetry at a scan rate of 1 mV s^−1^. The LbL multilayer buildup was studied using Quartz Crystal Microbalance (Q-Sense E4). Particle sizes and *z* potential were measured with a Zetasizer Nano Z (Malvern).

## Additional information

**How to cite this article:** Nyström, G. *et al*. Self-assembled three-dimensional and compressible interdigitated thin-film supercapacitors and batteries. *Nat. Commun*. 6:7259 doi: 10.1038/ncomms8259 (2015).

## Supplementary Material

Supplementary Information Supplementary Figures 1-6 and Supplementary References
