# Supplementary material for: Self-assembled three-dimensional and compressible interdigitated thin-film supercapacitors and batteries
Source: Nat Commun. 2015 May 29;6:7259. doi: 10.1038/ncomms8259 (PMC4458871; doi:10.1038/ncomms8259)
Supplement: Supplementary Information — Supplementary Figures 1-6 and Supplementary References [file ncomms8259-s1.pdf]

Supplementary Figures

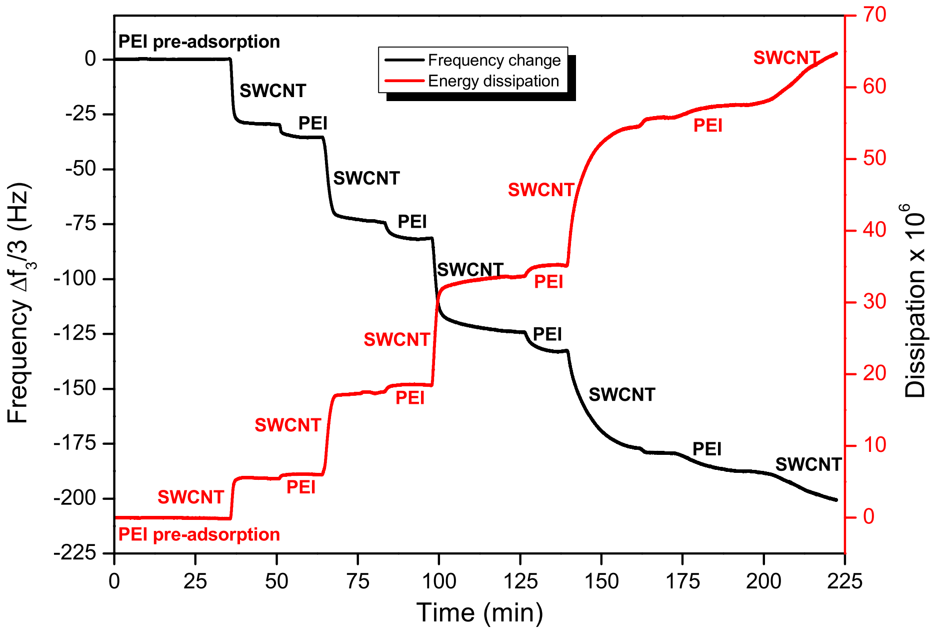

Supplementary Figure 1. 3D supercapacitor electrode build-up. Quartz crystal microbalance measurement showing the detailed buildup of the different layers of the supercapacitor electrode on a silica model surface. This indeed shows the multilayer buildup of the electrode but the time scale of this experiment is not related to the time required to form the real 3D electrodes completing the same assembly within 10 seconds.

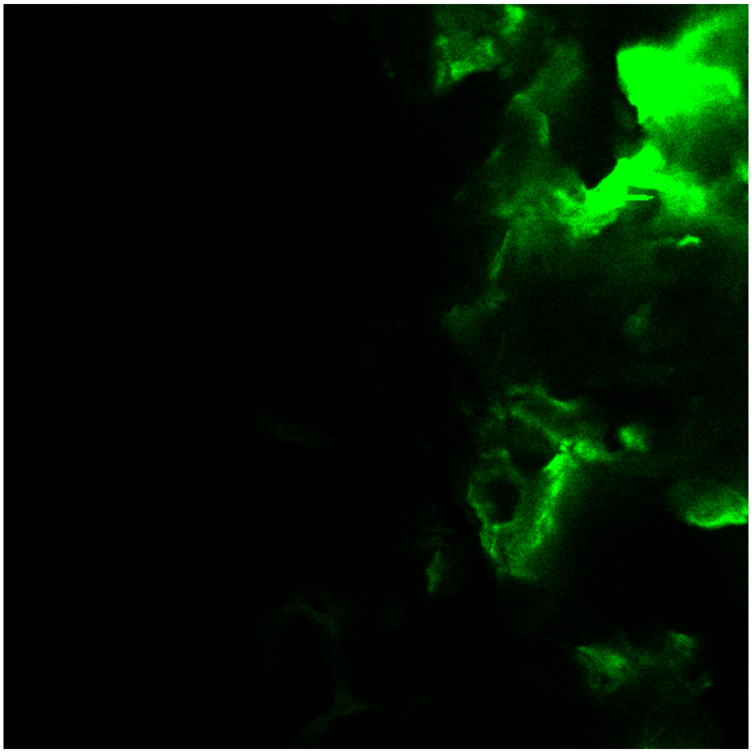

Supplementary Figure 2. 3D supercapacitor electrode masking. Fluorescence microscopy image of PEI / CNT coated aerogel where the dark regions have been masked with paraffin wax. The lack of fluorescence in these parts of the image shows that water and fluorescent dye does not penetrate into this part of the electrode.

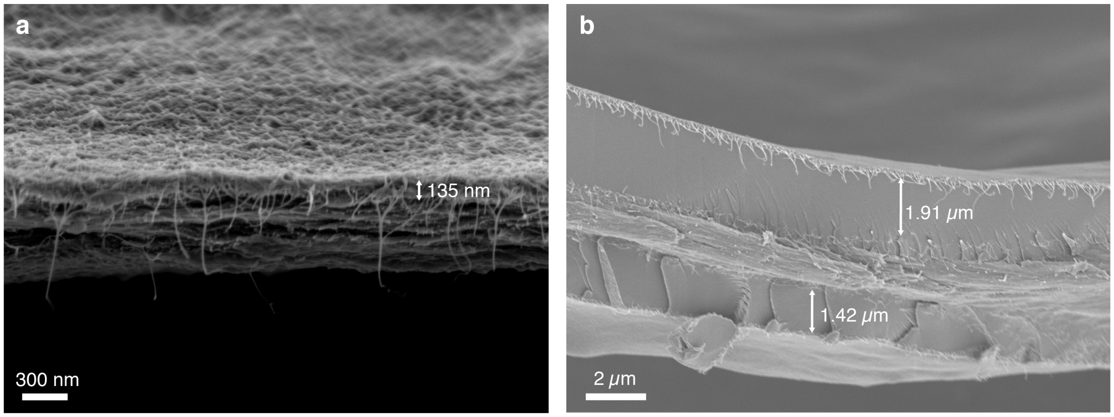

Supplementary Figure 3. 3D supercapacitor electrode and separator thickness estimations. (**a**) Examples of thickness estimations of the PEI/CNT electrode and (**b**) of the PEI/PAA separator by measurements in SEM images.

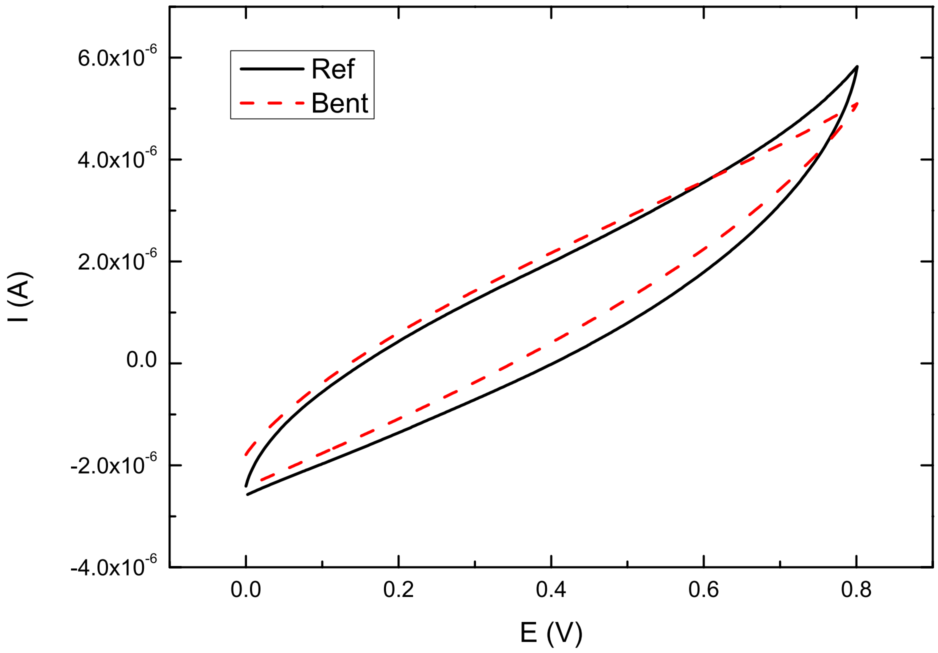

Supplementary Figure 4. Operation of 3D supercapacitor during bending. Cyclic voltammograms for the 3D supercapacitor operating without bending (black line) and under 90 degree bending (red dashed line).

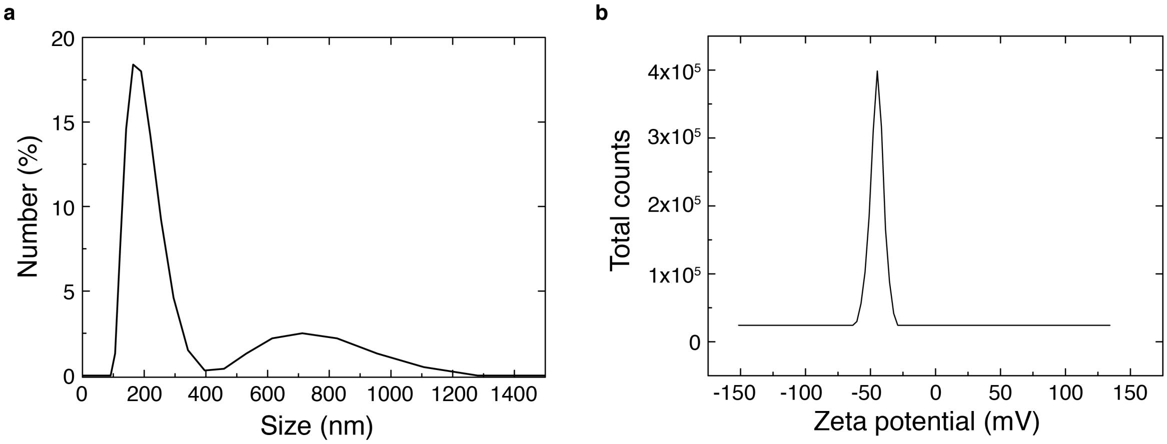

Supplementary Figure 5. Characterization of the hexacyanoferrate nanoparticles. (**a**) Particle size from dynamic light scattering and (**b**) z potential from electrophoretic mobility measurements for the copper hexacyanoferrate nanoparticles.

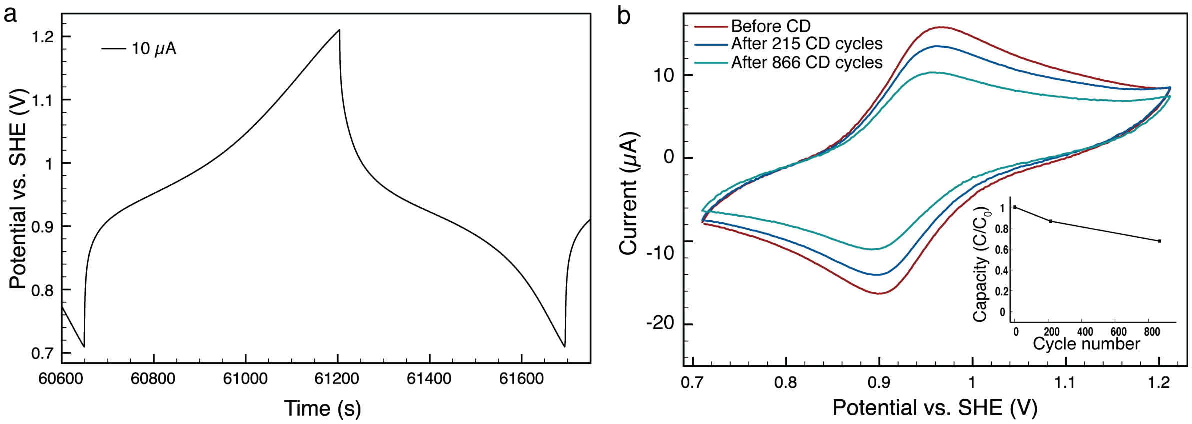

Supplementary Figure 6. Stability of the copper hexacyanoferrate cathode. (**a**) Typical galvanostatic charge and discharge curve at 10 μA charging current and (**b**) cyclic voltammogram at 1 mV s^{-1} before and after 215 and 866 cycles as well as the normalized capacity integrated from the CV reduction peak (inset).

SupplementaryReferences

1. Wessells, C. D., Huggins, R. A. & Cui, Y. Copper hexacyanoferrate battery electrodes with long cycle life and high power. *Nat. Commun.* **2,** 550 (2011).

2. Pasta, M., Wessells, C. D., Huggins, R. A. & Cui, Y. A high-rate and long cycle life aqueous electrolyte battery for grid-scale energy storage. *Nat. Commun.* **3,** 1149 (2012).

3. Wågberg, L. *et al*. The build-up of polyelectrolyte multilayers of microfibrillated cellulose and cationic polyelectrolytes. *Langmuir* **24,** 784–795 (2008).

4. Hamedi, M. *et al*. Nanocellulose Aerogels Functionalized by Rapid Layer-by-Layer Assembly for High Charge Storage and Beyond. *Angew. Chemie Int. Ed.* **52,** 12038–12042 (2013).
